# CRISPR-Based Diagnosis of Infectious and Noninfectious Diseases

**DOI:** 10.1186/s12575-020-00135-3

**Published:** 2020-09-14

**Authors:** Somayeh Jolany vangah, Camellia Katalani, Hannah A. Booneh, Abbas Hajizade, Adna Sijercic, Gholamreza Ahmadian

**Affiliations:** 1 Department of Industrial and Environmental Biotechnology, National Institute of Genetic Engineering and Biotechnology (NIGEB), Tehran, P.O.BOX: 14155-6343 Iran; 2 Department of Plant Biotechnology and Agricultural Science, Sari Agricultural Science and Natural Resource University, Sari, Iran; 3 Department of Genetics and Bioengineering, International Burch University, Francuske Revolucije bb, Ilidza, 71210 Sarajevo, Bosnia and Herzegovina; 4 Applied Microbiology Research Center, Systems Biology and Poisonings Institute, Baqiyatallah University of Medical Sciences, Tehran, Iran

**Keywords:** CRISPR-Cas, COVID-19, Diagnostic test, SHERLOCK, DETECTR, Single guide RNA (sgRNA)

## Abstract

Interest in CRISPR technology, an instrumental component of prokaryotic adaptive immunity which enables prokaryotes to detect any foreign DNA and then destroy it, has gained popularity among members of the scientific community. This is due to CRISPR’s remarkable gene editing and cleaving abilities. While the application of CRISPR in human genome editing and diagnosis needs to be researched more fully, and any potential side effects or ambiguities resolved, CRISPR has already shown its capacity in an astonishing variety of applications related to genome editing and genetic engineering. One of its most currently relevant applications is in diagnosis of infectious and non-infectious diseases. Since its initial discovery, 6 types and 22 subtypes of CRISPR systems have been discovered and explored. Diagnostic CRISPR systems are most often derived from types II, V, and VI. Different types of CRISPR-Cas systems which have been identified in different microorganisms can target DNA (e.g. Cas9 and Cas12 enzymes) or RNA (e.g. Cas13 enzyme). Viral, bacterial, and non-infectious diseases such as cancer can all be diagnosed using the cleavage activity of CRISPR enzymes from the aforementioned types. Diagnostic tests using Cas12 and Cas13 enzymes have already been developed for detection of the emerging SARS-CoV-2 virus. Additionally, CRISPR diagnostic tests can be performed using simple reagents and paper-based lateral flow assays, which can potentially reduce laboratory and patient costs significantly. In this review, the classification of CRISPR-Cas systems as well as the basis of the CRISPR/Cas mechanisms of action will be presented. The application of these systems in medical diagnostics with emphasis on the diagnosis of COVID-19 will be discussed.

## Introduction

Since the discovery of the clustered regularly interspaced short palindromic repeats (CRISPR) locus in *Escherichia coli* (*E. coli*) in 1987, [35] CRISPR has revolutionized both research and practical achievements in biology, particularly in the areas of genome editing and genetic engineering. CRISPR-associated genes (*cas* genes) were identified in 2002 [37] and further research has led to a deeper understanding of the structure as well as the function of CRISPR and CRISPR-associated (Cas) proteins. CRISPR-Cas systems are found in prokaryotic cells, both bacteria and archaea, where their main role is the protection of the organism against the introduction of exogenous DNA, such as plasmids and bacteriophages. These systems provide adaptive immunity to prokaryotes, which specifically destroy the foreign DNA molecules [6]. In the last few years, different CRISPR-Cas systems have been identified or engineered, each containing distinct characteristics and applications. These studies have generated many CRISPR toolboxes and many CRISPR-based technologies have emerged. The generation of in vivo and in vitro models [31, 63, 65] developing molecular tools for genome manipulation [48, 81] and antiviral and antibacterial drug development [1] are some of the main technologies based on CRISPR-Cas systems.

The main breakthrough within the field of genomic engineering was the demonstration of CRISPR-Cas in vitro genome editing [39]. After that, this technique was successfully exploited by different research groups for the manipulation of many genomes, including mice [86], *Drosophila* [8], *Caenorhabditis elegans* [21], humans [15, 17, 39, 69], zebrafish [32], bacteria [38], rice [88], etc.

**Fig. 1:**
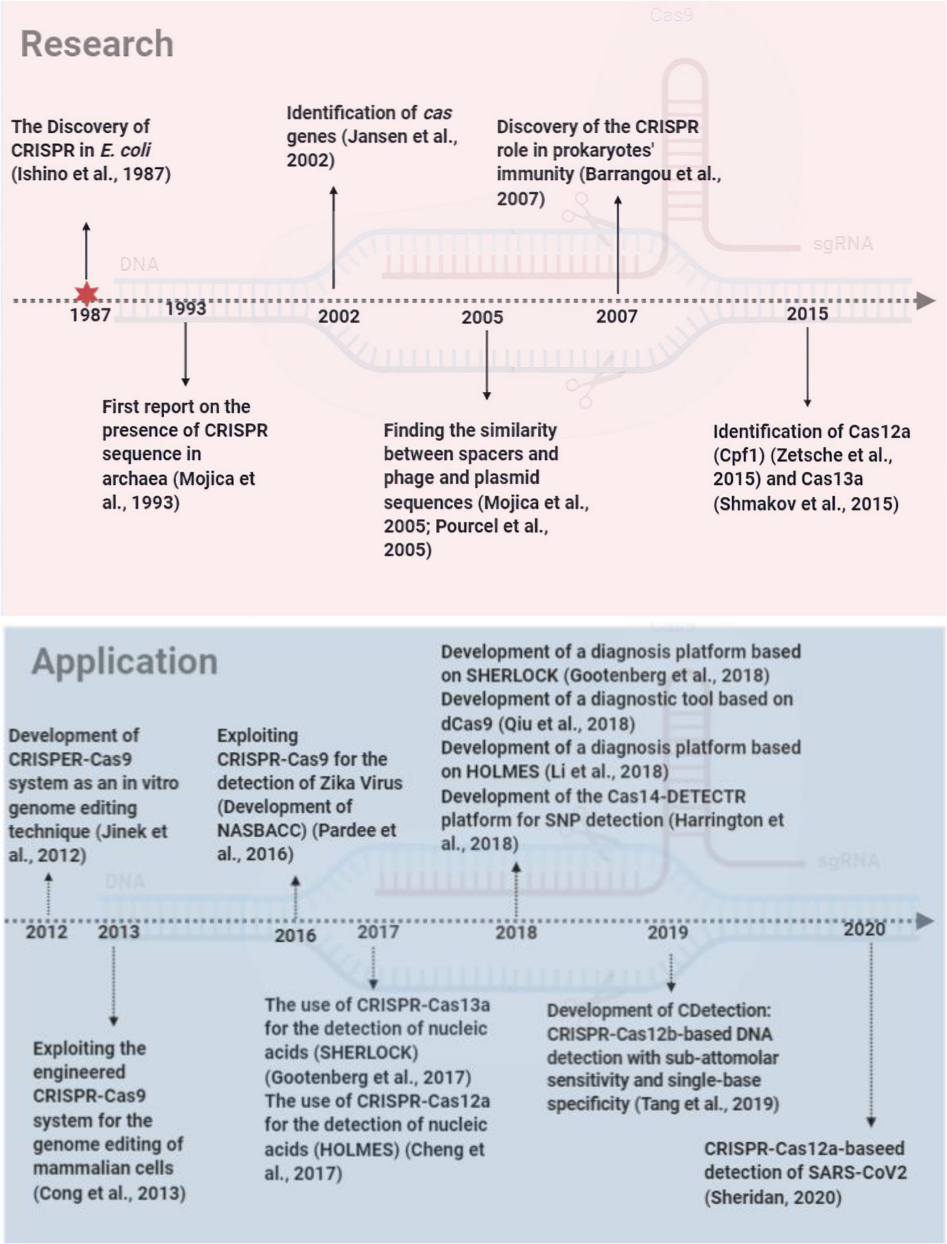
The timeline of the progresses of the CRSPR/Cas systems in research (pink chart) and applications (blue chart)

Because of the need for precise diagnosis in many disease situations, another important application of the CRISPR-Cas system has emerged: detection of diseases and microbes [33, 49]. Although robust and potent platforms were developed based on CRISPR-Cas9 [62, 90], the discovery of Cas13a (formerly C2c2) [70] and Cas12a (formerly Cpf1) [89] which both have collateral cleavage activity has revolutionized the field of nucleic acid detection. Cas13a is a single-component, RNA-guided and targeting enzyme, which is specific for ssRNA and collaterally cleaves neighbor non-targeted RNAs. In contrast, Cas12a is an RNA-guided, DNA-targeting enzyme which targets DNA and collaterally cleaves ssDNA (45). Different platforms have been developed based on these two proteins. Specific high sensitivity enzymatic reporter unlocking (SHERLOCK) was introduced by Gootenberg in 2017 [25] which exploits Cas13a for the detection of RNA molecules and a diagnostic platform based on this method was developed in 2018 [24]. At the same time, a Cas-12a-based diagnostic tool called one-hour low-cost multipurpose highly efficient system (HOLMES) was introduced in 2017 [14] and a diagnosis platform was described in 2018 [45]. A striking example of the power of these systems is the extremely fast development of a CRISPR-Cas-based diagnostic test which can rapidly and with a high sensitivity diagnose SARS-CoV-2, an emerging virus responsible for the COVID-19 pneumonia disease [11, 19, 49]. This demonstrated the high potential of CRISPR-Cas systems for the development of rapid detection of newly emerging diseases. Figure 1 shows the main milestones in the research and development of CRISPR-Cas systems with a focus on the role of these systems in medical diagnosis.

The biology of the CRISPR-Cas systems, their characteristics, and classification will be summarized in this review. Then, the application of CRISPR-Cas in the diagnosis of infectious and noninfectious diseases as well as the advantages and disadvantages of these systems will be described by relevant examples. Finally, the present status of the commercially available CRISPR-based diagnostics and the future perspectives of this field will be discussed.

## CRISPR-Cas Biology and Characteristics

**Fig. 2:**
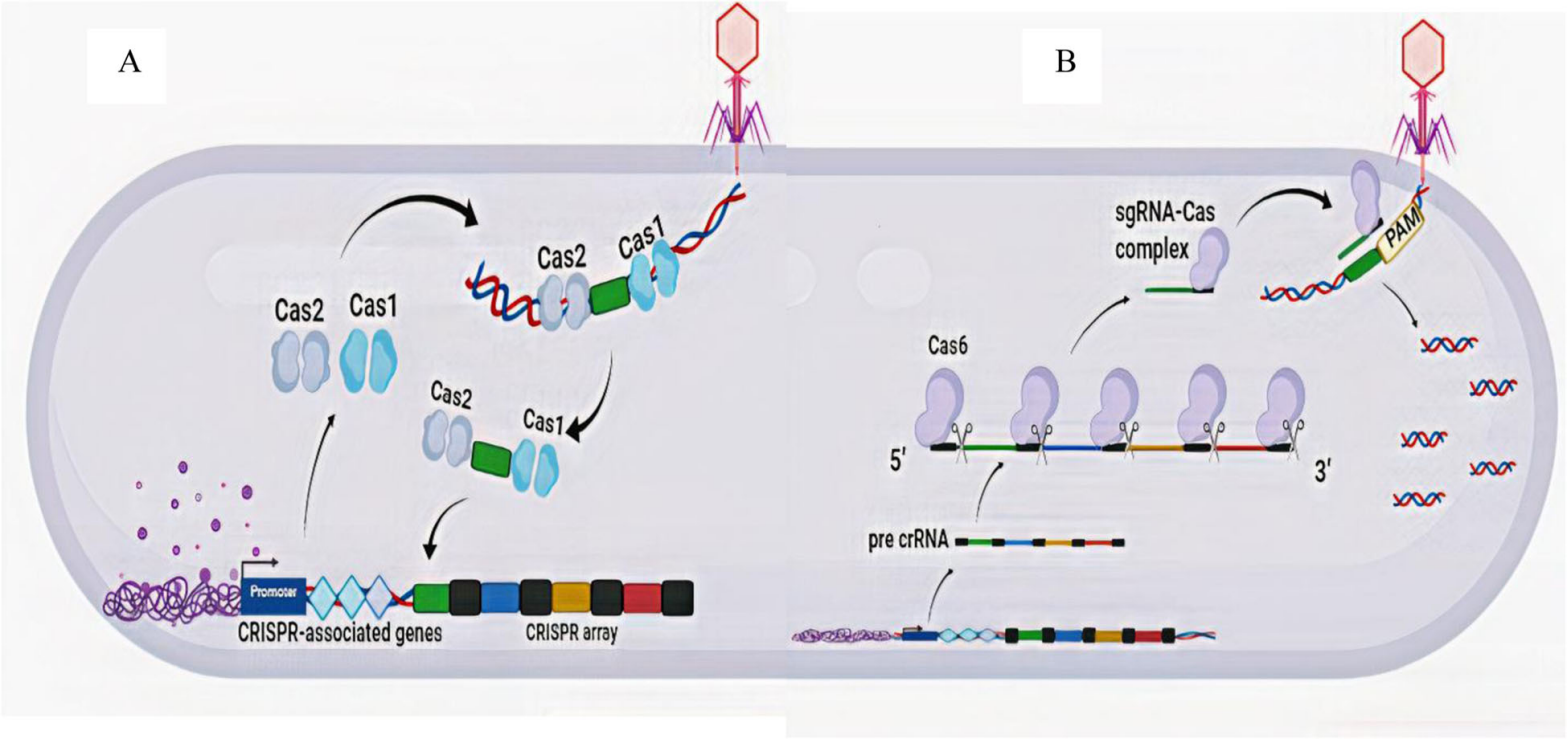
Biology of the CRISPR-Cas system. **a** Formation of CRISPR array occurs during adaptation process in which after injection of invader genome into the host cell, some Cas proteins like Cas1 and Cas2 recognize the protospacer sequence (green rectangle) and cut it and integrate it into host genome in CRISPR loci. **b** In crRNA maturation, a pre-crRNA is transcribed and Cas6 proteins attach to the 3′ end of repeat segments and cut the 5′ end of spacer fragments to construct the mature crRNA. In the interference process the mature crRNA-Cas protein complex recognizes and cuts the complementary sequence on the phage genome

CRISPR loci are composed of repeat sequences, with a length of about 20–40 bp, which are separated by unique interspaced 20–58 bp sequences called spacers [34]. A series of sequences are located downstream of the CRISPR locus which code for Cas proteins. At the time of their discovery, it was shown that some of these proteins were those previously thought to be involved in DNA repair [6]. Several different classes of Cas proteins with a variety of activities, including nuclease, helicase and polymerase activities which can be exploited for nucleic acid manipulation have been described. The CRISPR-Cas system is responsible for the adaptive immune response of prokaryotes [50]. Following the entry of viral DNA or plasmids into the cell for the first time, it will be degraded and inserted into specific sites between repeated sequences by Cas proteins and the CRISPR array will be organized (Fig. 2a).

However if the bacterial host does have a CRISPR system, these sequences are transcribed in the cell to generate CRISPR RNA (crRNA). Subsequently the crRNA accompanying Cas proteins forms an interfering complex. The complex, under crRNA guidance, directs formation of hydrogen bonding between the crRNA and the viral DNA that catalyzes the breaking apart of foreign DNA (Fig. 2b). Therefore, the infection essentially has ended before it starts. In fact, the CRISPR-Cas system acts like a bacterial immune system with the spacers in the CRISPR array presenting a history of old infections [34, 44].

## Classification of CRISPR-Cas Systems

Co-evaluation of specified anti-CRISPR proteins encoded by invading viruses furthermore progress the ability of innate immunity in archaeal and bacterial organisms, leading to remarkable diversity and fast adaptive evolution in Cas systems [44, 76]. Classification ofCRISPR-Cas systems was just emphasized on evolutionary relationships. Therefore combination of several criterion have recruited for classification schemes that are similar between conserved Cas proteins using: clustering and phylogenetic algorithm, architectural organization in the effector modules, the phylogeny of Cas1 and the presence of exclusive signature gene in the CRISPR-Cas loci. Although it’s harder than it seems, basically comparative genomic analysis and experimental data is essential for nomenclature and determining the molecular mechanisms of CRISPR-Cas systems [44, 52, 53]. The primary nomenclature of Cas genes based on the highest homology and frequency was termed on four names: Cas1, Cas2, Cas3 and Cas4 [37].

In recent years, the classification of CRISPR-Cas systems have developed considerably with the latest classification in 2020. Currently, based on the published classification, CRISPR-Cas systems are characterized in two major classes, which are further divided into six types and forty-eight subtypes [53].

Nearly 90% of the characterized CRISPR–cas loci located in Class 1 are most frequently found in fungal genomes with a small proportion found in bacteria. The known indicator of this class is the presence of heteromeric multiprotein effector complexes. Whilst Class 2 encompasses approximately 10% of all CRISPR–cas loci, it is more common in bacteria than other organisms, and is characterised by single multi-domain effector proteins [71].

### Class 1 Classification Scheme

**Fig. 3:**
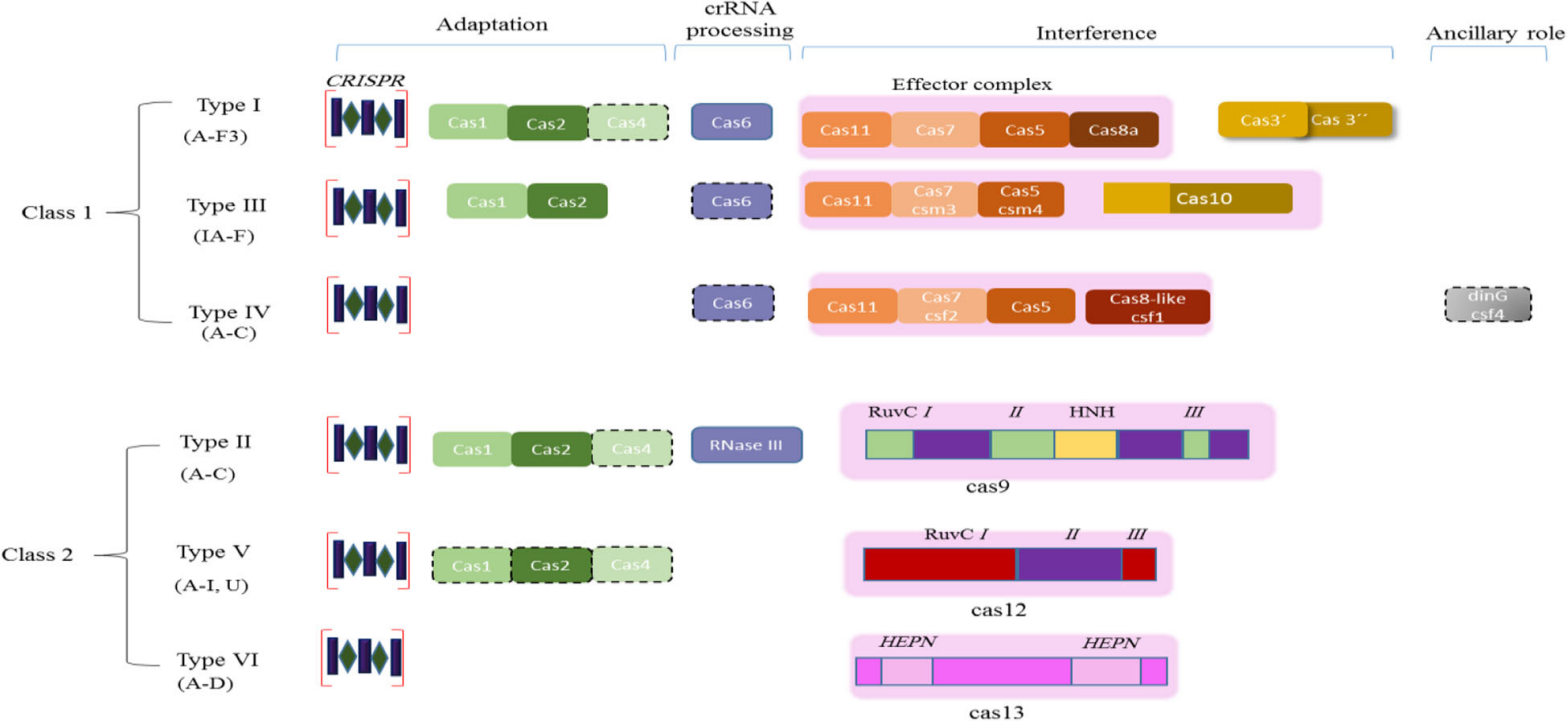
The recent classification and functional modules of CRISPR-Cas system. The effector complex in Class I composed of multiple Cas protein while in class II a single multidomain protein forms a crRNA-binding complex. The component that missed in some subtypes represented by dashed outlines. The figure is adapted and modified from Ref. [53]

Class 1 CRISPR-Cas systems consist of the three types, type I, III and IV systems, as well as 22 subtypes [53, 64]. The characteristic nucleases in Class I, which include enzymes Cas3, Cas10 and Cas8-like (*csf1*) are considered signature genes in types I, III, and IV, respectively (Fig. 3).

The target recognition and nucleic acid cleavage is carried out by effector modules that consist of Cas3, Cas5–8, Cas 10 and Cas 11 proteins [23, 73, 80]. The Repeat-Associated Mysterious Proteins (RAMPs), Cas7 and Cas5, exist in single or multiple copies and form the backbone of effector complexes in Class1 types. The large subunit of the effector modules in Class 1 consists of Cas8 and Cas10 in type I and III, respectively with Cas11 located with the small subunit [67, 82].

It has been shown that the type IV CRISPR-Cas system is a divergent derivative of types I and III. Typically, type IV lacks the adaptation complex. Despite a similar organization between effector modules and target recognition, there is minimal sequence conservation between types I, III and IV [60]. Also, there is high sequence divergence in the sequences of Cas5, Cas7 and cas6 in different subtypes [51].

Type I systems are further divided into nine subtypes I-A to I-G with derivatives of I-F1, I-F2 and I-F3 considered as separate subtypes. Phylogenetic trees demonstrating evolutionary relationships demonstrated that homology in Cas3΄ (a derivative of Cas3) exactly reflects the subtype classification [36]. The Cas3 protein is a helicase that acts to open DNA-DNA or DNA-RNA duplexes. The Cas 3 protein also contains an HD family endonuclease domain which participates in target DNA cleavage. This domain is located at the carboxy terminal end of Cas3 proteins of all subtypes except subtypes I-A and I-G [52].

The type III group of proteins is divided into subtypes from III-A to III-F. The signature gene Cas10 in this type encodes a protein containing a Palm domain, a form of RNA recognition motif (RRM). The Palm domain occupies the large subunit of crRNA–effector complexes and carries nucleic acid polymerase and cyclase activity [28]. The largest subunit in all type III proteins except subtype III-D is fused to an HD nuclease domain. One of the major activities mediated by the type III cas10-csm complex is RNA-guided cleavage of target DNA and its transcript that is performed using independent active sites in the complex [68].

The type IV group of proteins are less studied and consist of seven distinct variants. The majority of type IV systems lack any nucleolytic active sites, and would require use of host encoded restriction enzymes to degrade their targets [64]. Most type IV loci contain mobile genetic element (transposon-encoded) variants and are carried by plasmid-like elements that target other plasmids involved in plasmid competition [57, 64]. Some type IV proteins contain either a DinG family helicase or a small alpha helical protein.

### Expanding Class II Classification

Class 2CRISPR-Cas systems have a relatively simpler organization and distinct architecture than the other classes. Class 2 systems are divided into three types: types II, V, and VI, and 26 subtypes (Fig. 3) [70]. Classification of class 2 complexes has been recently been updated and expanded from an original 15 subtypes described in 2015 to now recognize 26 subtypes. This class of proteins has underpinned much of the great progress in recent years in fields of genome editing and rapid detection of infectious diseases, such as the recently emerged SARS-CoV-2 [87]. In subtype V-A and type VI proteins a large effector Cas protein is responsible for pre-crRNA processing while a non-Cas enzyme, bacterial RNase III, has robust activity as a processing module in type II and various type V subtypes. There are unique signature effector nucleases, with Cas9 for type II, Cas12 for type V and Cas13 for type VI, which plays a fundamental role in CRISPR-Cas classification [71].

Type II is the simplest and the most well-known CRISPR-Cas system containing four subtypes. The crRNA-effector complex in this type is a single multi-domain protein Cas9. It is possible that Cas 9 evolved from a mobile-genetic element unrelated to the CRISPR system, since the Cas9 signature gene on its own is not sufficient for classifying it as a particular CRISPR gene and Cas1 and Cas2 genes are utilized as auxiliary genes for identification of type II systems [16]. Cas9 requires two ancillary nuclease domains namely HNH and RuvC-like for cleavage the target DNA, and each nuclease only cleaves one strand of target DNA [70].

Until 2015, the type V system was considered as a presumed type, containing only one subgroup [52]. However, in the newest classification, the type V system was assigned the largest number of subtypes among all types related to CRISPR-Cas systems, with 17 derivative subtypes [53]. The cleavage of a double stranded DNA target by Type V systems is catalyzed by a RuvC-like nuclease domain, which is present in the Cas12 system [5, 22]. The Cas 12a, also known as Cpf1, provides a novel and sensitive platform named DNA endonuclease-targeted CRISPR trans reporter (DETECTR) used in various viral diagnostic assays that are considered below [13, 89].

The newest characterized CRISPR-Cas system is type VI that consists of five subtype variants. The effector complex in this type contains two HEPEN RNase domains involved in toxin-antitoxin modules that are associated in defense systems in bacterial or eukaryotic RNase L [3]. The effector protein Cas13a present in VI-A subtype acts as a kind of RNA-guided RNase, where Cas13a attaches to crRNA and forms a complex that cleaves ssRNA [25, 58]. A potent platform named SHERLOCK (specific high sensitivity enzymatic reporter unlocking) has been developed by Prof. Feng Zhang et al. as a potent platform for rapid and sensitive detection of many different infectious diseases, including COVID-19 [87].

## CRISPR-Cas System Mechanism

The CRISPR-Cas system functions through three different steps: adaptation (or spacer acquisition), crRNA maturation, and interference (Figs. 2 and 3).

### Adaptation

In the adaptation stage, Cas1 and Cas2 are the two main protein components which play roles in three main types (I, II, and III) of CRISPR-Cas systems. Both of these proteins are dimers that can form a complex together in order to undergo acquisition of foreign DNA [30]. Cas1 has both nuclease and integrase activity and can cut the viral genome and integrate a specific piece of the viral genetic element into the spacer DNA, whereas Cas2 is an endoribonuclease that mainly cuts RNAs [30, 75]. Since the subtype I-E of the CRISPR-Cas system of *E. coli* has been well-studied and characterized, it has become the best model for analysis of the adaptation mechanism of type I systems. In CRISPR-Cas type I and II systems, a short sequence named the protospacer-adjacent motif (PAM) exists in the foreign genome that leads Cas1 and Cas2 to recognize the protospacer flanked region. In subtype I-E of *E. coli*, in the presence of PAM, Cas1 and Cas2 recognize the adjacent sequence to the PAM. Cas1 and Cas2 dimers cut the PAM-adjacent sequence. After adjusting the size of the protospacer, the presence of the integrated host factor (IHF) protein is required for integrating the spacer into the host genome. By bending the host DNA near the insertion site, IHF guides Cas1 and Cas2 to the correct position of the CRISPR array for integrating the spacer [30, 75]. However, in the CRISPR-Cas system of *Streptococcus pyogenes* (subtype II-A), other Cas proteins including Cas9, Csn2 (whose function is related to Cas1 and Cas2 in DNA acquisition process [85], trans-activating CRISPR RNA (tracrRNA) and the leader-anchoring site (LAS) element in place of IHF are required for correct integration of the spacer sequence by Cas1 and Cas2 [75].

### crRNA Maturation

In the crRNA maturation step, transcription begins in the CRISPR array. The RNA transcribed by this process is termed pre-crRNA and contains complementary sequences of the repeats at the 3′ end and the spacers at the 5′ end. The maturation process of pre-crRNA occurs differently in various types of CRISPR-Cas systems [75]. In type I CRISPR-Cas systems, palindromic repeat sequences of pre-crRNA located at the 3′ end of the transcript, transform these parts of pre-crRNA into a hairpin-like structure. Following pre-crRNA transcription, Cas6 endoribonuclease attaches to the hairpin-like structure and cuts the 5′ end of the spacer sequence adjacent to that hairpin-like sequence. At the end of this process, there are several mature crRNAs that enable a Cas6 protein to remain bound to each one. An exception to this is seen with processes involving types I-A and I-B where the repeat sequences are not palindromic and Cas6 releases the crRNA [30]. This product is a ribonucleoprotein that identifies a specific phage genome [29].

In type II CRISPR-Cas systems, tracrRNA binds to the repeat sequence on the crRNA and transforms the 3′end of crRNA into a double-strand RNA. This double-strand RNA is called single guide RNA (sgRNA). In this type, RNase III and Cas9 catalyze the cleavage of pre-crRNA that yield to maturation crRNAs [12].

In type III systems, a dimer of Cas6 cleaves the 3′end of the repeat sequences adjacent to the spacers within the pre-crRNA. Once the cleavage process is complete the mature crRNA is then released.

crRNA maturation in type IV CRISPR systems is unknown at present. Cas12 and Cas13 carry out the cleavage of pre-crRNA and maturation of crRNA in types V and VI, respectively [75].

### Interference

During the final step of interference, type-specific Cas proteins together with crRNA form a complex that recognizes and cuts the invader’s genome. In types I and II, the PAM plays an important role to increase the specificity of recognizing the invader’s genetic elements because not only can the crRNA identify the phage’s DNA sequence but the Cas enzyme can distinguish the PAM sequence [83].

In type I systems, interference occurs through a complex involving crRNA and Cas6, which acts as a scaffold for attaching other Cas proteins (including Cas5, Cas7, Cas8, and Cas11) to form CRISPR-associated complex for antiviral defense (Cascade). This in turn recognizes the invader’s genome. Moreover, recognition of the PAM sequence by Cas8e as the large subunit of Cascade increases the specificity of target recognition. After binding between crRNA of complex and target DNA the complex recruits Cas3 which functions as the nuclease that cleaves the non-target DNA strand to make an intermediated product. The ultimate cleavage of target DNA seems to occur by another nuclease provided either by the host cell or by utilizing the cascade-independent activity of Cas3 [7, 30].

In type II interference, a complex consisting of sgRNA and Cas9 recognizes the target DNA and cleaves it. This particular types has been utilized extensively for the purpose of gene editing in addition to diagnostics [16].

Type III interference is similar to that of type I systems where a complex of Cas proteins (involving Cas5, Cas7, Cas10, and Cas11) named Csm (subtype III-A) and Cmr (subtype III-B) accompanied by crRNA, recognizes the RNA that is transcribed from target DNA. After binding to this single-strand RNA, cleavage is carried out by subunits of Cas7. Alternatively, Cas10 can catalyze dsDNA cleavage and by transforming ATP into cyclic AMP as a second messenger, can activate the Csm6 protein which is an RNase. Csm6 then cleaves remaining RNAs in a nonspecific manner [30].

Type IV interference has not undergone characterization [30]. Interference in type V systems is divided into three subtypes: A, B, and C, in which the effector proteins in interference processes are Cas12a, Cas12b, and Cas12c, respectively. Similar to Cas9, Cas12b and Cas12c need the tracrRNA for their interfering activity while Cas12a does not. Cas12 proteins have a collateral nuclease activity which has been used in diagnostic applications such as the DNA endonuclease-targeted CRISPR trans reporter (DETECTR) method. During the interference step, recognizing the target double-strand DNA (dsDNA) and PAM sequence is accomplished by crRNA and Cas12, respectively. After binding this complex to the target, Cas12 cuts the dsDNA and by its collateral nuclease activity also cuts surrounding single-strand DNAs nonspecifically. Both of these nuclease activities are catalyzed by the RuvC domain of Cas12a protein [46].

In type VI system interference, Cas13, which has higher eukaryotes and prokaryotes nucleotide (HEPN)-binding domains [30], which has collateral nuclease activity like Cas12 acts as the effector protein. This system is utilized in specific high sensitivity enzymatic reporter unlocking (SHERLOCK) technology. Cas13 does not require tracrRNA and PAM. The target of the Cas13-crRNA complex is single-strand RNAs (ssRNAs). In addition to crRNA guidance, the Cas13-crRNA complex requires a protospacer flanking site (PFS) for binding to the complementary ssRNA. PFS is an analogue of the PAM sequences on the RNA targets and is required for Cas13a activity in *Leptotrichia shahii* [30]. After binding, the complex cleaves the target and non-target ssRNAs [46, 75].

## CRISPR as a Diagnostic Tool

Using the principle that nucleic acids are effective biomarkers for diseases, CRISPR-based diagnostic methods rely primarily on identifying a certain sequence associated with a disease and then cleaving it in order to produce a readable signal. Examples of target sequences include oncogenic mutation sequences or viral and bacterial sequences derived from the infectious agent. The goal of CRISPR systems is to identify the specific pathogens, as well as to repair alleles that cause disease through specific DNA sequence editing at exact locations on the chromosome [20]. The goal of CRISPR systems is to identify the specific pathogens, as well as to repair alleles that cause disease through specific DNA sequence editing at exact locations on the chromosome [79].

Various properties of the CRISPR system have led to development of various diagnostic methods. While some tests make use of both the identification and cleavage of the target, other tests function based singularly on the guide RNA and Cas protein identification of the target [79]. Identification of the CRISPR system in pathogenic bacteria has become a useful diagnostic tool, due to the CRISPR being a part of most bacterial defense systems. Certain diagnostic methods based on these mechanisms are: serotyping/subtyping utilizing CRISPR, diagnostic assay based on single guide RNA (SgRNA), and another method of diagnostic assay based on CRISPR interference (dCas9) [79].

The rapid, sensitive, specific, accurate, cheap, and reliable, features of CRISPR-based diagnostic tools provide huge potential for applications in a wide range of areas [41, 46, 84]. They have the capacity not only for detection of pathogens during an epidemic, but also in cancer diagnosis, single-nucleotide polymorphisms (SNPs) identification, and genetic disease detection [41]. The highly sensitive nature of CRISPR diagnostic tests is derived from the fact that most are able to utilize fluorescent probes which are highly sensitive. This specificity arises from the binding to the target via Watson-Crick base pairing between DNA-RNA or RNA-RNA. The tests can proceed at a rapid pace since it is not necessary to culture isolates or extract genomic DNA [79].

### CRISPR-Based Diagnosis of Viruses

**Fig. 4:**
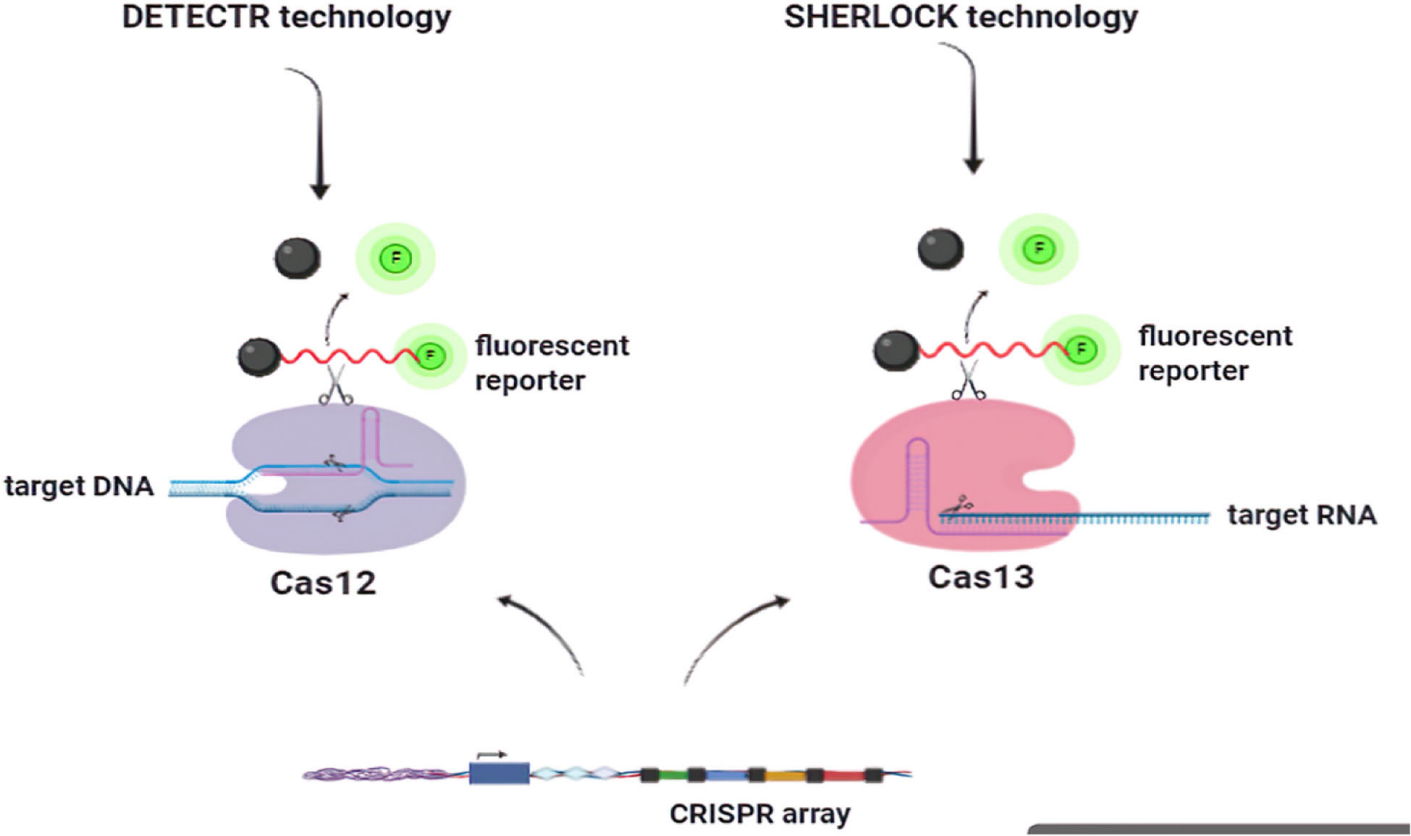
Cas12 and Cas13 Cleavage Activity. In the DETECTR technology, after binding the Cas12-crRNA complex to its target (dsDNA) the collateral nuclease activity of the Cas12 leads to cleavage of the reporter molecule nonspecifically after which the fluorescent signal is detectable. In the SHERLOCK technology, Cas13a guided by the single CRISPR RNA (crRNA) to cleave ssRNA or mRNA and the same process occurs

The most widely explored area for CRISPR-based diagnostic systems is within the field of viral infection. Several researchers have developed methods based on the CRISPR-Cas12a and Cas13a families, dubbed DETECTR and SHERLOCK, respectively [10, 42]. As indicated above and shown in Fig. 4, DETECTR uses the Type V Cas12a enzyme to directly bind to DNA targets in a three-stage process: a guide RNA first directs the Cas12a enzyme to a double-stranded sequence of DNA within a specified viral genome [13]. Once bound to its viral genetic target, a single-stranded DNA molecule bound to a quencher molecule and a reporter fluorophore are cleaved indiscriminately by the Cas12a enzyme [42]. This “collateral” cleavage is detected as a fluorescent signal released from the fluorophore and quencher [13]. The primary advantage of the DETECTR method lies in its high sensitivity, as it is able to detect a single molecule of viral particle within a microliter of sample [42].

In the SHERLOCK approach, detection occurs by binding and cleaving RNA indiscriminately through the use of crRNA targets via the Type VI Cas13a enzyme [24, 42]. A targeting molecule with an attached fluorophore binds to the target RNA and cleaves it in a collateral manner as seen in Fig. 4, causing a fluorescence signal in the presence of specific sequences, which can then be detected and analyzed to confirm the presence of virus nucleic acid [10]. SHERLOCK has been explored significantly for its uses in viral detection and diagnosis since its initial creation, and researchers have further optimized the method, producing the simplified and more specific SHERLOCKv2 protocol [24]. Improvements include the addition of multiplexing which was accomplished through identifying orthogonal sequencing ability by optimizing enzymes from Cas13a and Cas13b families, resulting in the ability to identify four differing RNA target sequences within a single reaction through fluorescence reporting [10]. Cas13 enzymes were also combined with the supplemental CRISPR-associated Csm6 enzyme, which more than tripled sensitivity [24]. DETECTR and SHERLOCK methods can be applied to diagnose a significant array of viruses in both laboratory and clinical settings [10]. The DETECTR method has been utilized significantly for diagnosis of human papillomavirus (HPV), although it can be applied to theoretically any virus [10]. Both SHERLOCK and DETECTR methods can be coupled with recombinase polymerase amplification (RPA) to enhance amplification and detection of viral material [42, 56]. Furthermore, the SHERLOCK protocol can be optimized for diagnosis of human immunodeficiency virus (HIV), which continues to be a viral pathogen of significant concern worldwide [10].

To make the SHERLOCK procedure even more efficient, the Heating Unextracted Diagnostic Samples to Obliterate Nucleases (HUDSON) protocol was created in order to detect viral genetic material from bodily fluids including urine, blood and its isolates, and saliva [56]. HUDSON protocol researchers found that conserved regions within the genetic material of these viruses can be identified using universal-flavivirus RPA, as well as crRNAs specific to a given viral species [56]. SHERLOCK and HUDSON protocols can also be applied to any virus, but previous testing focused on diagnosis of flaviviruses such as Zika, Dengue, West Nile, and yellow fever viruses [24, 56].

Of significant acute interest to scientists currently is how CRISPR methods can be applied to diagnosis of the novel coronavirus (SARS-CoV-2), an emerging pathogen which has infected over 12.9 million people and killed over 500,000 to date [40, 89]. SARS-CoV-2 is an enveloped RNA-based virus of the *Coronaviridae* family, and it causes mild to severe symptoms across various demographics. Of additional concern is the substantial incubation period, as a person can have the virus but remain asymptomatic for up to two weeks before showing symptoms [59]. The DETECTR method has been used for detection of this virus and in the applications described focuses on identifying the presence of the N and E gene variants specific to SARS-CoV-2. A positive result is generated if both genes are detected, and the procedure has been optimized to exclude false positives resulting from related coronaviruses [11]. The proposed SHERLOCK method generates a positive result for SARS-CoV-2 when the S and Orflab gene sequences are detected [90].

### Bacterial Diagnosis by CRISPR System

CRISPR-based procedures and methods have been greatly explored for their use against viruses, but they can also be applied for bacterial diagnosis, especially in identifying antimicrobial drug resistant bacteria. The CRISPR-Cas9 system is among the major systems used for molecular diagnostics, facilitating detection and characterization of diseases, including those caused by bacterial infection [84]. A single guide RNA (sgRNA) directs the endonuclease Cas9 to DNA sequence which has been targeted, and initiates site-specific manipulation [79]. The Type II CRISPR/Cas9 system is an extensively used DNA-editing method, as a result of the ability to design CRISPR-guided nucleases in this system easily and relatively quickly [20, 27].

One method, dubbed FLASH (Finding Low Abundance Sequences by Hybridization), uses Cas9 enzyme recombination along with multiplex guide RNAs for precise identification of a pathogen by eliminating background sequences, and the Cas9 system cleaves target sequences into fragments ideal for next generation sequencing [66]. FLASH and its associated software tool FLASHit was used to design a Cas9 enzyme set which would target a total of 3624 bacterial genetic sequences associated with antimicrobial drug resistance. The method was used to test drug resistance of *S. aureus* cultured isolates, but also had significant application in direct testing of clinical cases, including in patients with MRSA infections and vancomycin-resistant *E. faecium* [66].

Besides the FLASH method for diagnosis drug-resistant bacterial infections, CRISPR has usability for rapid *Mycobacterium tuberculosis* (Mtb) testing using the Cas12a system. The procedure for Mtb diagnosis uses RPA followed by detection through Cas12a optimized enzymes [2]. Additionally, CRISPR techniques were used in 2011 during an outbreak of enterohemorrhagic *E. coli*. The bacterial strain causing the outbreak was the hybrid strain STEC O104:H4, and CRISPR-based testing focused on identifying the O104:H4 locus specific to the hybrid with a 99.06% sensitivity rate [18]. It is also worth noting that CRISPR systems can be used for treatment of antimicrobial drug-resistant bacterial infections using bacteriophages or vectors, although a full consideration of this potential function of CRISPR is beyond the scope of this review [4, 84].

### CRISPR-Based Diagnosis of Non-infectious Diseases

Since its discovery, the CRISPR/Cas9 system has been recognized as an applicable tool for the purpose of identifying oncogenes and other mediators of cancer, and has become integrated into cancer research. Currently, CRISPR technology is used to investigate the genetic mechanisms in almost all areas of cancer [78].

The CRISPR/Cas9 system can also be utilized for drug resistance blocking, as it can successfully identify synergistic gene interactions [78]. Furthermore, post-treatment gene expression changes as well as pinpointed genes associated with resistance to targeted drugs can be revealed by functional genome-screening approaches using the CRISPR system. This offers the potential for offering new insights into cancer development with identification of new precision therapy biomarkers [78]. Moreover, determining sensitive genes through the use of genetic diagnostics is crucial for cancer prevention. The CRISPR-based diagnostic system SHERLOCK which uses Cas13 has been established and successfully used for such needs [41, 78].

## Pros and Cons of CRISPR-Based Diagnosis Systems

Two types of diagnostic tests exist for the detection of viral pathogens: those that target host antibodies produced in response to viral infection and those that target the virus genome directly. Tests based on antibody reactions (also known as serological tests) blood sample acquisition from suspected patients several days after viral transmission to give their immune system enough time to produce antibodies [11, 47].

On the other hand, nucleic acid-based tests that rely on qRT-PCR to amplify reverse-transcribed viral RNA focus on identifying the virus directly present in samples. Thus, these tests can detect viruses in patient samples even before symptoms start. Therefore, the nucleic acid-based tests are more useful over antibody-based tests to counter the spread of a virus. Therefore, point-of-care and rapid testing fights the spread of highly contagious viruses, as it allows infected individuals to be rapidly identified, so they can be contact-traced and isolated (Table 1) [11, 26].

**Table 1:** Pros and cons of CRISPR-based assay for infectious disease. Adapted from James P. Broughton  et. al., 2020, Nature biotechnology, CRISPR–Cas12-based detection of SARS-CoV-2

	SARS-CoV-2 DETECTR, RT–LAMP/Cas12	CDC SARS-CoV-2 qRT–PCR
Target	E gene and N gene^a^	N gene (three amplicons, N1, N2 and N3)
Sample control	RNase P	RNase P
LoD	10 copies per μl input	1 copy per μl input^b^ and 3.2 copies per μl input^c^
Assay reaction time (approximate)	30–40 min	120 min
Assay sample-to-result time (approximate)	45 min (with manual RNA extraction)	4 h (including RNA extraction)
Assay results	Qualitative	Quantitative
Assay components	RT–LAMP (62 °C, 20–30 min) Cas12 (37 °C, 10 min) Lateral flow strip (RT, 2 min; no additional time if using fluorescence readout)	UDG digestion (25 °C, 2 min), reverse transcription (50 °C, 15 min), denature (95 °C, 2 min) amplification, (95 °C, 3 s; 55 °C 30 s; 45 cycles)
Bulky instrumentation required	No	Yes
US FDA EUA approval	Pending clinical validation	Yes

a: E gene primers target the same amplicon region as in the WHO protocol; N gene primers target the same N2 amplicon region as in the CDC protocol. UDG, uracil-DNA glycosylase

b: Limit of detection confirmation CDC 2019-nCoV Real-Time RT-PCR Diagnostic Panel with QIAGEN QIAmp DSP Viral RNA Mini Kit6

C: Limit of detection confirmation of the CDC 2019-nCoV Real-Time RT-PCR Diagnostic Panel with QIAGEN EZ1 DSP6

The disadvantages associated with current diagnostic strategies such as serological tests and nucleic acid-based tests, led the researchers to develop CRISPR-based techniques DETECTR and SHERLOCK assays [13, 25] which are distinguished by targeting DNA and RNA, respectively. The two CRISPR-based strategies meet these criteria in addition to offering unique multiplexing abilities [1, 26]. Particularly, the CRISPR-based DETECTR assay is as accurate as qRT–PCR but is a more rapid approach as well. Therefore, the limitations that are mentioned for qRT–PCR assays including availability of personal protective equipment, [9] extraction kits, and reagents, as well as sample collection and RNA extraction methods, also exist for the CRISPR-based diagnosis assay early stages. However, the CRISPR-based approach has some more advantages over qRT–PCR including rapid turnaround time, isothermal signal amplification that prevents the need for thermocycling, single nucleotide target specificity, no requirement for complex laboratory setup, and integration with accessible reporting formats including lateral flow strips [33]. CRISPR systems have a unique ability to be quickly reorganized to diagnose infectious diseases caused by emerging viruses, showcased by the rapid development of the DETECTR assay for the diagnosis of SARS-COV-2. A quick diagnostic CRISPR-based solution is able to identify the mutated strains of coronavirus family members with high fidelity. Because the rapid and timely detection of viruses, including SARS-CoV-2, in an infected person is of great importance for epidemiological studies and subsequent treatment development of diagnostic strategies to control the spread are necessary. Allied with this is the importance of being able to detect asymptomatic infections and the appearance of mutant strains of the virus as they arise. Another important point regarding coronavirus is that the viral load can fluctuate during the day and at different stages of infection, therefore a qRT–PCR test could be negative at the time when the viral load is low but it does not exclude infection and a more accurate test is needed [33].

Because the sequences used to design guide RNA in CRISPR are selected from the conserved region between strains, even if a virus undergoes mutations, theCRISPR-Cas -based diagnosis test will be able to detect it. As the test is based on the diagnosis of a viral genome, it can be used at any stage of infection, especially in the early stages of the incubation period, without the need for additional confirmatory tests. One of the main drawbacks of the current available tests is that they cannot detect the virus immediately after infection and need time to increase the load of the virus; therefore false-negative results can be obtained [55].

However, one of the main disadvantages ofCRISPR-Cas system is the non-specific binding of sgRNA to the genome of the organism under study (off-target phenomenon) which in turn, leads to the poor signaling and misinterpretation of the results.

In order to reduce the effects of off-target CRISPR-Cas complex, sgRNAs must be designed using specialized bioinformatics tools to select the best ones. Since cleavage sites are usually located in areas rich in uracil of ssRNA or ssRNA loops or ssRNA junctions with dsRNA inside the predicted target sequence, folding prediction software can be used to address these cases. Another problem that might be faced when using CRISPR-based methods is that binding to the target sequence may be affected by its secondary structure and the presence of RNA-binding proteins which is especially important when using CRISPR for genome modification [41, 55].

A significant advantage of both SHERLOCK and DETECTR is that they can be completed more quickly than RT-PCR tests (~ 30 min versus > 1 h) due to their use of isothermal amplification technologies, as these methods negate the need for denaturing DNA by using strand-displacing DNA polymerases. In addition, both of these tests can be adapted for detection through the use of lateral flow dipsticks, making bulky thermocycling and/or detection equipment unnecessary. Turnaround time reduction and limited equipment requirements make CRISPR diagnostics increasingly effective candidates for rapid diagnostic assays. Additionally, CRISPR-based DETECTR assay can be supplemented with microfluidic- or SPR-based (Surface Plasmon Resonance) detection systems to develop a portable rapid test applicable at the site of the patient [61, 91], a module which is not possible with other tests, including qRT-PCR, which need expensive instrumentation.

The most important advantage ofCRISPR-Cas diagnostics systems is their multiplexing capability. It is possible to design pathogen-specific crRNAs from a conserved region of the pathogen genome. The multiplex diagnostic capability can discriminate between multiple viral pathogens or even different serotypes of the viruses in the same sample [56].

Compared to the previous approaches such as meganucleases and zinc finger nucleases CRISPR-based methods offer a more cost-effective and easier methods of genome detection or manipulations. The CRISPR-Cas system was selected as the primary revolutionary method in genome editing research due to its easier manipulation, simpler steps, and lower cost. By improving treatment methods, this system might contribute to human health by fast and efficient detection of many diseases [43].

In the near future, CRISPR-Cas kits consisting of various Cas proteins and a variety of sgRNA with different structures and molecules for different purposes are expected to become more popular tools. In addition, new categories of targeting nucleases similar to CRISPR-Cas, whose function depends on Watson and Cricket pairs, can complement current systems. Generally, it is clear that a bright future awaits CRISPR-based diagnostic and genome engineering techniques.

## Commercially Available Diagnostic Tools Based on CRISPR

Since its emergence as a genome-editing and diagnostic tool, many companies have developed commercially available kits for utilization of CRISPR. Among the most notable are the DETECTR and SHERLOCK systems mentioned previously [10]. DETECTR is sold by Mammoth Biosciences, and their diagnostic kits are programmable for a wide array of viral and bacterial infections, as well as cancer diagnosis [54]. The SHERLOCK researchers have made great strides in developing an affordable, time-efficient method using paper-strip lateral flow readout assays. These types of tests have been developed for Zika and Dengue virus [25]. Additionally, with the addition of the HUDSON protocol, the SHERLOCK protocol bypasses the purification and dilution steps normally necessary for sequence analysis and analyzes human samples directly, an ability of significant interest to medical and laboratory practitioners needing to provide rapid diagnosis for viral infections and field-deployable diagnostic kits [42, 56]. Both the DETECTR and SHERLOCK methods have been proposed as diagnostic methods for SARS-CoV-2, and the protocols designed specifically for SARS-CoV-2 detection can be accomplished in an hour or less, and at relatively low cost [7, 90]. While there is significant interest in the Type V Cas12a and Type VI Cas13-based systems, the Type II CRISPR-Cas 9 systems still have much to offer as gene editing and diagnostic tools. Cas9 systems have been used for diagnosis of bacterial and viral infections, and are offered by several labs and companies commercially, such as Sigma-Aldrich (Table 2) [72, 79].

**Table 2:** Commercially available diagnostic tools based on CRISPR

Diagnostic System/Instrument	CRISPR Type	Manufacturing Company
DETECTR Diagnostic Tests	V	Mammoth Biosciences
SHERLOCK Diagnostic Tests	VI	SHERLOCK
CRISPR/Cas9 Products for Gene Editing	II	Sigma-Aldrich

One of the interesting emerging topics surrounding CRISPR is the possibility of its utilization by “DIY scientists” [74]. There are companies who are offering simple CRISPR kits at prices as low as 200 USD, making it possible for individuals with virtually no prior training to attempt CRISPR-based experiments [77]. Admittedly, this is being used more for amateur gene editing than diagnostics, but there is serious potential for emerging CRISPR techniques and methods to come from more amateur sources in the future [74].

## Conclusion

The CRISPR-Cas system was adapted from a natural gene editing process in bacteria in which the CRISPR palindromic repeats play an important role in microbial immunity. Since the initial discovery of the CRISPR system within bacteria, researchers have utilized and reprogrammed it to allow efficient genome editing in various species. It has been shown that the CRISPR system has the potential to completely change medicine as well as showing promising applications in treating different human and plant diseases, developing disease models and in the area of biofuels. The fast evolution of CRISPR-Cas systems for cell and molecular biology research have been due to the efficiency, relative simplicity, and versatility of the system.

One of the exciting applications of the CRISPR-Cas biology is in the field of infectious diseases, understanding the fundamentals of the host and microbe interactions and especially to further development of accurate and rapid diagnostic tools able to detect small genomic fragments. Using specifically designed synthetic sgRNA, CRISPR systems can be used to detect nucleic acids involved in both infectious and non-infectious diseases and in the development of portable diagnostic tests to advance the identification, treatment, and prevention of infectious disease.

Researchers are working continuously to design new diagnostic tests based on the CRISPR gene-editing system. For instance, the Cas12 and Cas13 enzymes that, unlike the Cas9 enzyme, detect target sequences and cut the fragments frequently, a behavior which is not desirable in gene editing, have an advantage in diagnosis of specific DNA and RNA fragments, as these cuts can release the signals which can be visualized and detected. This approach can help diagnose a wide range of bacterial and viral infections early to allow implementation of treatments in a timely manner to more effectively prevent the spread of those diseases. Further diagnosis of infectious diseases using methods such as PCR requires high expertise and sophisticated equipment, all of which are limited especially in underdeveloped countries. The CRISPR-based diagnostic system makes it possible to diagnose infections with the same accuracy of conventional methods but with the lower cost.

We have covered in this review the recent emerging CRISPR applications in basic and applied research that could routinely be integrated into daily practice in the near future and further studies will focus specifically on optimizing this technology.

## Data Availability

Not applicable for this study.

## Abbreviations

ssRNAs: single-strand RNAs
ssDNAs: single-strand DNAs
RT-PCR: Reverse-transcription Polymerase Chain Reaction
CRISPR: Clustered regularly interspaced short palindromic repeats
Cas: CRISPR-associated proteins
SHERLOCK: Specific high sensitivity enzymatic reporter unlocking
DETECTR: DNA endonuclease-targeted CRISPR trans reporter
PAM: Protospacer-adjacent motif
SgRNA: single guide RNA
FLASH: Finding Low Abundance Sequences by Hybridization
HUDSON: Heating Unextracted Diagnostic Samples to Obliterate Nucleases

## References

[1] Abbott TR, Dhamdhere G, Liu Y, Lin X, Goudy L, Zeng L, et al. Development of CRISPR as an antiviral strategy to combat SARS-CoV-2 and influenza. Cell. 2020;181(4):865–76. 10.1016/j.cell.2020.04.020 PMC7189862 32353252

[2] Ai JW, Zhou X, Xu T, Yang M, Chen Y, He GQ, Pan N, Cai Y, Li Y, Wang X, Su H (2019). CRISPR-based rapid and ultra-sensitive diagnostic test for mycobacterium tuberculosis. Emerg Microbes Infect.

[3] Anantharaman V, Makarova KS, Burroughs AM, Koonin EV, Aravind L (2013). Comprehensive analysis of the HEPN superfamily: identification of novel roles in intra-genomic conflicts, defense, pathogenesis and RNA processing. Biol Direct.

[4] Bakhrebah M, Nassar M, Alsuabeyl M, Zaher W, Meo S (2018). CRISPR technology: new paradigm to target the infectious disease pathogens. Eur Rev Med Pharmacol Sci.

[5] Bao W, Jurka J (2013). Homologues of bacterial TnpB_IS605 are widespread in diverse eukaryotic transposable elements. Mob DNA.

[6] Barrangou R, Fremaux C, Deveau H, Richards M, Boyaval P, Moineau S (2007). CRISPR provides acquired resistance against viruses in prokaryotes. Science..

[7] Barrangou R (2013). CRISPR-Cas systems and RNA-guided interference. Wiley Interdiscip Rev RNA.

[8] Bassett AR, Tibbit C, Ponting CP, Liu J-L (2013). Highly efficient targeted mutagenesis of Drosophila with theCRISPR-Cas 9 system. Cell Rep.

[9] Bauchner H, Fontanarosa PB, Livingston EH (2020). Conserving supply of personal protective equipment—a call for ideas. Jama.

[10] Bhattacharyya RP, Thakku SG, Hung DT (2018). Harnessing CRISPR effectors for infectious disease diagnostics. ACS Infect Dis.

[11] Broughton JP, Deng X, Yu G, Fasching CL, Servellita V, Singh J, Miao X, Streithorst JA, Granados A, Sotomayor-Gonzalez A, Zorn K. CRISPR–Cas12-based detection of SARS-CoV-2. Nat Biotechnol. 2020;16:1–5. 10.1038/s41587-020-0513-4 PMC9107629 32300245

[12] Charpentier E, van der Oost J, White MF (2013). crRNA biogenesis. CRISPR-Cas systems: Springer.

[13] Chen JS, Ma E, Harrington LB, Da Costa M, Tian X, Palefsky JM (2018). CRISPR-Cas12a target binding unleashes indiscriminate single-stranded DNase activity. Science..

[14] Cheng QX (2017). An application of a Cas protein, and a method and kit for detecting a target nucleic acid molecule.

[15] Cho SW, Kim S, Kim JM, Kim J-S (2013). Targeted genome engineering in human cells with the Cas9 RNA-guided endonuclease. Nat Biotechnol.

[16] Chylinski K, Makarova KS, Charpentier E, Koonin EV (2014). Classification and evolution of type II CRISPR-Cas systems. Nucleic Acids Res.

[17] Cong L, Ran FA, Cox D, Lin S, Barretto R, Habib N (2013). Multiplex genome engineering usingCRISPR-Cas systems. Science.

[18] Delannoy S, Beutin L, Burgos Y, Fach P (2012). Specific detection of enteroaggregative hemorrhagic Escherichia coli O104:H4 strains by use of the CRISPR locus as a target for a diagnostic real-time PCR. J Clin Microbiol.

[19] Ding X, Yin K, Li Z, Liu C. All-in-One dual CRISPR-cas12a (AIOD-CRISPR) assay: a case for rapid, ultrasensitive and visual detection of novel coronavirus SARS-CoV-2 and HIV virus. bioRxiv. 2020. 10.1038/s41467-020-18575-6 PMC7501862 32948757

[20] Foss DV, Hochstrasser ML, Wilson RC (2019). Clinical applications of CRISPR-based genome editing and diagnostics. Transfusion..

[21] Frøkjær-Jensen C (2013). Exciting prospects for precise engineering of Caenorhabditis elegans genomes withCRISPR-Cas 9. Genetics.

[22] Gomes-Filho JV, Zaramela LS, VCdS I, Baliga NS, Vêncio RZ, Koide T (2015). Sense overlapping transcripts in IS 1341-type transposase genes are functional non-coding RNAs in archaea. RNA Biol.

[23] Gong B, Shin M, Sun J, Jung C-H, Bolt EL, van der Oost J (2014). Molecular insights into DNA interference by CRISPR-associated nuclease-helicase Cas3. Proc Natl Acad Sci.

[24] Gootenberg JS, Abudayyeh OO, Kellner MJ, Joung J, Collins JJ, Zhang F (2018). Multiplexed and portable nucleic acid detection platform with Cas13, Cas12a, and Csm6. Science..

[25] Gootenberg JS, Abudayyeh OO, Lee JW, Essletzbichler P, Dy AJ, Joung J (2017). Nucleic acid detection with CRISPR-Cas13a/C2c2. Science..

[26] Gulei D, Raduly L, Berindan-Neagoe I, Calin GA (2019). CRISPR-based RNA editing: diagnostic applications and therapeutic options. Expert Rev Mol Diagn.

[27] Guo L, Sun X, Wang X, Liang C, Jiang H, Gao Q (2020). SARS-CoV-2 detection with CRISPR diagnostics. Cell Discov.

[28] Hale CR, Zhao P, Olson S, Duff MO, Graveley BR, Wells L (2009). RNA-guided RNA cleavage by a CRISPR RNA-Cas protein complex. Cell..

[29] Hatoum-Aslan A, Maniv I, Marraffini LA (2011). Mature clustered, regularly interspaced, short palindromic repeats RNA (crRNA) length is measured by a ruler mechanism anchored at the precursor processing site. Proc Natl Acad Sci U S A.

[30] Hille F, Richter H, Wong SP, Bratovic M, Ressel S, Charpentier E (2018). The biology of CRISPR-Cas: backward and forward. Cell..

[31] Hou P, Chen S, Wang S, Yu X, Chen Y, Jiang M (2015). Genome editing of CXCR4 byCRISPR-Cas 9 confers cells resistant to HIV-1 infection. Sci Rep.

[32] Hruscha A, Krawitz P, Rechenberg A, Heinrich V, Hecht J, Haass C (2013). EfficientCRISPR-Cas 9 genome editing with low off-target effects in zebrafish. Development.

[33] Huang C-H, Lee K-C, Doudna JA (2018). Applications of CRISPR-Cas enzymes in cancer therapeutics and detection. Trends in cancer.

[34] Ishino Y, Krupovic M, Forterre P. History of CRISPR-Cas from Encounter with a Mysterious Repeated Sequence to Genome Editing Technology. J Bacteriol. 2018;200(7). 10.1128/JB.00580-17 PMC5847661 29358495

[35] Ishino Y, Shinagawa H, Makino K, Amemura M, Nakata A (1987). Nucleotide sequence of the iap gene, responsible for alkaline phosphatase isozyme conversion in Escherichia coli, and identification of the gene product. J Bacteriol.

[36] Jackson RN, Lavin M, Carter J, Wiedenheft B (2014). Fitting CRISPR-associated Cas3 into the helicase family tree. Curr Opin Struct Biol.

[37] Jansen R, JDv E, Gaastra W, Schouls LM (2002). Identification of genes that are associated with DNA repeats in prokaryotes. Mol Microbiol.

[38] Jiang W, Bikard D, Cox D, Zhang F, Marraffini LA (2013). RNA-guided editing of bacterial genomes using CRISPR-Cas systems. Nat Biotechnol.

[39] Jinek M, East A, Cheng A, Lin S, Ma E, Doudna J (2013). RNA-programmed genome editing in human cells. elife.

[40] Johns Hopkins University. COVID-19 Dashboard by the Center for Systems Science and Engineering (CSSE) at Johns Hopkins University (JHU). 2020.

[41] Khambhati K, Bhattacharjee G, Singh V (2019). Current progress in CRISPR-based diagnostic platforms. J Cell Biochem.

[42] Kocak D, Gersbach C (2018). From CRISPR scissors to virus sensors. Nature.

[43] Konermann S, Lotfy P, Brideau NJ, Oki J, Shokhirev MN, Hsu PD (2018). Transcriptome engineering with RNA-targeting type VI-D CRISPR effectors. Cell.

[44] Koonin EV, Makarova KS, Zhang F (2017). Diversity, classification and evolution of CRISPR-Cas systems. Curr Opin Microbiol.

[45] Li S-Y, Cheng Q-X, Wang J-M, Li X-Y, Zhang Z-L, Gao S (2018). CRISPR-Cas12a-assisted nucleic acid detection. Cell Discov.

[46] Li Y, Li S, Wang J (2019). Liu G.CRISPR-Cas systems towards next-generation biosensing. Trends Biotechnol.

[47] Liu J, Liao X, Qian S, Yuan J, Wang F, Liu Y (2020). Community transmission of severe acute respiratory syndrome coronavirus 2, Shenzhen, China, 2020.

[48] Liu X, Hao R, Chen S, Guo D, Chen Y (2015). Inhibition of hepatitis B virus by theCRISPR-Cas 9 system via targeting the conserved regions of the viral genome. J Gen Virol.

[49] Lucia C, Federico P-B, Alejandra GC. An ultrasensitive, rapid, and portable coronavirus SARS-CoV-2 sequence detection method based on CRISPR-Cas12. bioRxiv. 2020.

[50] Makarova KS, Grishin NV, Shabalina SA, Wolf YI, Koonin EV (2006). A putative RNA-interference-based immune system in prokaryotes: computational analysis of the predicted enzymatic machinery, functional analogies with eukaryotic RNAi, and hypothetical mechanisms of action. Biol Direct.

[51] Makarova KS, Haft DH, Barrangou R, Brouns SJ, Charpentier E, Horvath P (2011). Evolution and classification of the CRISPR–Cas systems. Nat Rev Microbiol.

[52] Makarova KS, Wolf YI, Alkhnbashi OS, Costa F, Shah SA, Saunders SJ (2015). An updated evolutionary classification of CRISPR–Cas systems. Nat Rev Microbiol.

[53] Makarova KS, Wolf YI, Iranzo J, Shmakov SA, Alkhnbashi OS, Brouns SJ, et al. Evolutionary classification of CRISPR–Cas systems: a burst of class 2 and derived variants. Nat Rev Microbiol. 2019:1–17. 10.1038/s41579-019-0299-x PMC8905525 31857715

[54] Mammoth Biosciences. Diagnostics - The CRISPR-based detection platform. Mammoth Biosci. 2020; [cited 1 July 2020]. Available from: https://mammoth.bio/diagnostics/.

[55] Metsky HC, Freije CA, Kosoko-Thoroddsen T-SF, Sabeti PC, Myhrvold C. CRISPR-based surveillance for COVID-19 using genomically-comprehensive machine learning design. BioRxiv. 2020.

[56] Myhrvold C, Freije CA, Gootenberg JS, Abudayyeh OO, Metsky HC, Durbin AF (2018). Field-deployable viral diagnostics using CRISPR-Cas13. Science..

[57] Newire E, Aydin A, Juma S, Enne VI, Roberts AP. Identification of a Type IV CRISPR-Cas system located exclusively on IncHI1B/IncFIB plasmids in Enterobacteriaceae. bioRxiv. 2020;11(1937). 10.3389/fmicb.2020.01937 PMC7434947 32903441

[58] O'Connell MR (2019). Molecular mechanisms of RNA targeting by Cas13-containing type VI CRISPR–Cas systems. J Mol Biol.

[59] Ortiz-Prado E, Simbaña-Rivera K, Gómez-Barreno L, Rubio-Neira M, Guaman LP, Kyriakidis NC, Muslin C, Jaramillo AM, Barba-Ostria C, Cevallos-Robalino D, Sanches-SanMiguel H (2020). Clinical, molecular and epidemiological characterization of the SARS-CoV2 virus and the coronavirus disease 2019 (COVID-19), a comprehensive literature review. Diagn Microbiol Infect Dis.

[60] Özcan A, Pausch P, Linden A, Wulf A, Schühle K, Heider J (2019). Type IV CRISPR RNA processing and effector complex formation in Aromatoleum aromaticum. Nat Microbiol.

[61] Pan Y, Zhang D, Yang P, Poon LL, Wang Q (2020). Viral load of SARS-CoV-2 in clinical samples. Lancet Infect Dis.

[62] Pardee K, Green AA, Takahashi MK, Braff D, Lambert G, Lee JW, Ferrante T, Ma D, Donghia N, Fan M, Daringer NM (2016). Rapid, low-cost detection of Zika virus using programmable biomolecular components. Cell.

[63] Park RJ, Wang T, Koundakjian D, Hultquist JF, Lamothe-Molina P, Monel B (2017). A genome-wide CRISPR screen identifies a restricted set of HIV host dependency factors. Nat Genet.

[64] Pinilla-Redondo R, Mayo-Muñoz D, Russel J, Garrett RA, Randau L, Sørensen SJ (2020). Type IV CRISPR–Cas systems are highly diverse and involved in competition between plasmids. Nucleic Acids Res.

[65] Platt RJ, Chen S, Zhou Y, Yim MJ, Swiech L, Kempton HR (2014). CRISPR-Cas9 knockin mice for genome editing and cancer modeling. Cell..

[66] Quan J, Langelier C, Kuchta A, Batson J, Teyssier N, Lyden A, Caldera S, McGeever A, Dimitrov B, King R, Wilheim J (2019). FLASH: a next-generation CRISPR diagnostic for multiplexed detection of antimicrobial resistance sequences. Nucleic Acids Rese.

[67] Reeks J, Naismith JH, White MF (2013). CRISPR interference: a structural perspective. Biochem J.

[68] Samai P, Pyenson N, Jiang W, Goldberg GW, Hatoum-Aslan A, Marraffini LA (2015). Co-transcriptional DNA and RNA cleavage during type III CRISPR-Cas immunity. Cell..

[69] Schwank G, Koo BK, Sasselli V, Dekkers JF, Heo I, Demircan T, Sasaki N, Boymans S, Cuppen E, van der Ent CK, Nieuwenhuis EE (2013). Functional repair of CFTR byCRISPR-Cas 9 in intestinal stem cell organoids of cystic fibrosis patients. Cell Stem Cell.

[70] Shmakov S, Abudayyeh OO, Makarova KS, Wolf YI, Gootenberg JS, Semenova E (2015). Discovery and functional characterization of diverse class 2 CRISPR-Cas systems. Mol Cell.

[71] Shmakov S, Smargon A, Scott D, Cox D, Pyzocha N, Yan W (2017). Diversity and evolution of class 2 CRISPR–Cas systems. Nat Rev Microbiol.

[72] Sigma-Aldrich. CRISPR-Cas 9 Products and Services. Merck. 2020; [cited 29 June 2020]. Available from: https://www.sigmaaldrich.com/catalog/product/sigma/crispr?lang=en&region=ME&gclid=Cj0KCQjwoub3BRC6ARIsABGhnyYDx3aJs1fGoi7Y6tDKDw05uFW-DdUTxSzLrzmxmTgyzS4c6a049nUaAjzTEALw_wcB.

[73] Sinkunas T, Gasiunas G, Fremaux C, Barrangou R, Horvath P, Siksnys V (2011). Cas3 is a single-stranded DNA nuclease and ATP-dependent helicase in theCRISPR-Cas immune system. EMBO J.

[74] Sneed A. Mail-order CRISPR kits allow absolutely anyone to hack DNA. Sci Am. 2017;2.

[75] Strich JR, Chertow DS. CRISPR-Cas Biology and Its Application to Infectious Diseases. J Clin Microbiol. 2019;57(4). 10.1128/JCM.01307-18 PMC6440769 30429256

[76] Takeuchi N, Wolf YI, Makarova KS, Koonin EV (2012). Nature and intensity of selection pressure on CRISPR-associated genes. J Bacteriol.

[77] The ODIN (2020). DIY HUMAN CRISPR GUIDE. The ODIN.

[78] Tian X, Gu T, Patel S, Bode AM, Lee MH (2019). Dong Z.CRISPR-Cas 9–an evolving biological tool kit for cancer biology and oncology. NPJ Precision Oncol.

[79] Uppada V, Gokara M, Rasineni GK (2018). Diagnosis and therapy with CRISPR advanced CRISPR based tools for point of care diagnostics and early therapies. Gene..

[80] Van Der Oost J, Westra ER, Jackson RN, Wiedenheft B (2014). Unravelling the structural and mechanistic basis of CRISPR–Cas systems. Nat Rev Microbiol.

[81] Van Diemen FR, Kruse EM, Hooykaas MJ, Bruggeling CE, Schürch AC, van Ham PM (2016). CRISPR-Cas 9-mediated genome editing of herpesviruses limits productive and latent infections. PLoS Pathog.

[82] Venclovas Č (2016). Structure of Csm2 elucidates the relationship between small subunits of CRISPR-Cas effector complexes. FEBS Lett.

[83] Vorontsova D, Datsenko KA, Medvedeva S, Bondy-Denomy J, Savitskaya EE, Pougach K (2015). Foreign DNA acquisition by the I-F CRISPR-Cas system requires all components of the interference machinery. Nucleic Acids Res.

[84] Wang W, Hou J, Zheng N, Wang X, Zhang J (2019). Keeping our eyes on CRISPR: the" atlas" of gene editing. Cell Biol Toxicol.

[85] Wei Y, Terns RM, Terns MP (2015). Cas9 function and host genome sampling in type II-A CRISPR-Cas adaptation. Genes Dev.

[86] Wu Y, Liang D, Wang Y, Bai M, Tang W, Bao S, Yan Z, Li D, Li J (2013). Correction of a genetic disease in mouse via use of CRISPR-Cas9. Cell Stem Cell.

[87] Xiang X, Qian K, Zhang Z, Lin F, Xie Y, Liu Y, Yang Z. CRISPR-cas systems based molecular diagnostic tool for infectious diseases and emerging 2019 novel coronavirus (COVID-19) pneumonia. J Drug Target, 1–5. Advance online publication. 2020. 10.1080/1061186X.2020.1769637. 10.1080/1061186X.2020.1769637 PMC7265108 32401064

[88] Xie K, Yang Y (2013). RNA-guided genome editing in plants using a CRISPR–Cas system. Mol Plant.

[89] Zetsche B, Gootenberg JS, Abudayyeh OO, Slaymaker IM, Makarova KS, Essletzbichler P (2015). Cpf1 is a single RNA-guided endonuclease of a class 2 CRISPR-Cas system. Cell..

[90] Zhang F, Abudayyeh OO, Gootenberg JS (2020). A protocol for detection of COVID-19 using CRISPR diagnostics. A protocol for detection of COVID-19 using CRISPR diagnostics.

[91] Zou L, Ruan F, Huang M, Liang L, Huang H, Hong Z (2020). SARS-CoV-2 viral load in upper respiratory specimens of infected patients. N Engl J Med.

