# Correction to: CRISPR-Based Diagnosis of Infectious and Noninfectious Diseases

**DOI:** 10.1186/s12575-020-00136-2

**Published:** 2020-11-07

**Authors:** Somayeh Jolany vangah, Camellia Katalani, Hannah A. Boone, Abbas Hajizade, Adna Sijercic, Gholamreza Ahmadian

**Affiliations:** 1 Department of Industrial and Environmental Biotechnology, National Institute of Genetic Engineering and Biotechnology (NIGEB), Tehran , P.O.BOX: 14155-6343 , Iran; 2 Department of Plant Biotechnology and Agricultural Science, Sari Agricultural Science and Natural Resource University, Sari, Iran; 3 Department of Genetics and Bioengineering, International Burch University, Francuske Revolucije bb, Ilidza, 71210 Sarajevo, Bosnia and Herzegovina; 4 Applied Microbiology Research Center, Systems Biology and Poisonings Institute, Baqiyatallah University of Medical Sciences, Tehran, Iran

**Correction to: Biol Proced Online 22, 22 (2020)**

10.1186/s12575-020-00135-3

In the original publication of this article [1], there was an error in the article, the authors opted to correct the last name of co-author Hannah Abigail Boone from Booneh to Boone. The original article has been corrected.
